# Synergistic effects of p53 activation via MDM2 inhibition in combination with inhibition of Bcl-2 or Bcr-Abl in CD34^+^ proliferating and quiescent chronic myeloid leukemia blast crisis cells

**DOI:** 10.18632/oncotarget.5890

**Published:** 2015-09-29

**Authors:** Bing Z. Carter, Po Yee Mak, Duncan H. Mak, Vivian R. Ruvolo, Wendy Schober, Teresa McQueen, Jorge Cortes, Hagop M. Kantarjian, Richard E. Champlin, Marina Konopleva, Michael Andreeff

**Affiliations:** ^1^ Section of Molecular Hematology and Therapy, Department of Leukemia, The University of Texas MD Anderson Cancer Center, Houston, Texas, USA; ^2^ Department of Leukemia, The University of Texas MD Anderson Cancer Center, Houston, Texas, USA; ^3^ Department of Stem Cell Transplantation and Cellular Therapy, The University of Texas MD Anderson Cancer Center, Houston, Texas, USA

**Keywords:** blast crisis CML, quiescent CD34+ cells, Nutlin3a, TKI, Bcl-2

## Abstract

The Bcr-Abl tyrosine kinase regulates several Bcl-2 family proteins that confer resistance to apoptosis in chronic myeloid leukemia (CML) cells. Given p53's ability to modulate the expression and activity of Bcl-2 family members, we hypothesized that targeting Bcr-Abl, Bcl-2, and p53 concomitantly could have therapeutic benefits in blast crisis (BC) CML and in quiescent CML CD34^+^ cells that are insensitive to tyrosine kinase inhibitors (TKI). We examined the effects of the MDM2 inhibitor nutlin3a and its combination with the dual Bcl-2 and Bcl-xL inhibitor ABT-737, and the Bcr-Abl inhibitor nilotinib on BC CML patient samples. We found that in quiescent CD34^+^ progenitors, p53 expression is significantly lower, and MDM2 is higher, compared to their proliferating counterparts. Treatment with nutlin3a induced apoptosis in bulk and CD34^+^CD38^−^ cells, and in both proliferating and quiescent CD34^+^ progenitor CML cells. Nutlin3a synergized with ABT-737 and nilotinib, in part by inducing pro-apoptotic, and suppressing anti-apoptotic, Bcl-2 proteins. Nilotinib inhibited the expression of Bcl-xL and Mcl-1 in BC CML cells. These results demonstrate that p53 activation by MDM2 blockade can sensitize BC CML cells, including quiescent CD34^+^ cells, to Bcl-2 inhibitor- and TKI-induced apoptosis. This novel strategy could be useful in the therapy of BC CML.

## INTRODUCTION

The development of Bcr-Abl tyrosine kinase inhibitors (TKIs) for the treatment of chronic myeloid leukemia (CML) is the most successful molecular targeted therapy to date. Indeed, TKIs were approved as front-line therapy for patients with chronic phase CML. However, when patients progresses to blast crisis (BC), CML cells becomes less responsive to TKIs [1] and few options are available for these patients. Hence, BC CML is essentially an incurable disease with a significant clinical impact.

The current standard of therapy for CML is inactive against quiescent CML stem/progenitor cells [2–6], which make up a small fraction of the CD34^+^ cell population that is responsible for disease recurrence upon drug discontinuation [7–9]. Furthermore, in addition to the stem/progenitor cells derived from hematopoietic stem cells as described in chronic phase CML, the committed granulocyte-macrophage progenitors in BC CML gain an abnormal self-renewal capacity [10] that is responsible for the rapid cell expansion in BC [11]. Thus, a number of strategies have been developed pre-clinically, or are under development clinically, to overcome these problems by combining TKIs with various other targeted agents [2, 12–17].

The oncogenic Bcr-Abl fusion protein promotes leukemic cell growth and survival through multiple mechanisms. For example, in addition to triggering aberrant cell proliferation, a number of anti-apoptotic Bcl-2 proteins such as Mcl-1 and Bcl-xL are regulated by Bcr-Abl tyrosine kinase signaling, which can confer apoptosis resistance to CML cells [18, 19]. Conversely, inhibition of anti-apoptotic Bcl-2 proteins can sensitize CML progenitor cells to TKIs and other agents [12, 20, 21]. We previously demonstrated that inhibition of Bcl-2/Bcl-xL by ABT-737 alone can induce apoptosis in CD34^+^ CML BC progenitor cells, and this effect is further enhanced by combining ABT-737 with imatinib [14]. This finding suggested an important role for anti-apoptotic Bcl-2 family members in the survival of CML progenitor cells.

*TP53* is a key tumor suppressor gene, and the modulation of Bcl-2 family proteins is a principal mechanism of p53-mediated cell death. p53 not only transcriptionally activates pro-apoptotic Bcl-2 family members [22–24], it also antagonizes anti-apoptotic Bcl-2 and Bcl-xL in the cytosol and directly contributes to mitochondrial-mediated apoptosis [25, 26]. In recent years, substantial pre-clinical evidence has confirmed the activation of p53 by MDM2 (the E3 ligase for p53 [27]) blockade as a promising cancer therapy strategy. Indeed, reports from our group and others have shown that the activation of p53 via MDM2 inhibition induces cell death and enhances efficacy of chemotherapeutic agents in hematological malignancies [28–32]. Lastly, overexpression of MDM2 has been reported to correlate with nutlin3a sensitivity in both AML and ALL [28, 32].

Although *TP53* mutation rate is known to increase with CML disease progression, a 30% reported rate of BC CML cell mutations is markedly lower than the frequency of *TP53* mutations reported in solid tumors [33]. Furthermore, increased MDM2 expression in BC CML compared to latent/chronic phase CML has been reported [34]. Interestingly, MDM2 has been shown to be regulated by Bcr-Abl and may play an essential role in the survival effects of Bcr-Abl signaling [35]. It has been further reported that p53 activation by SIRT1 inhibition, in combination with imatinib increased the killing of CML progenitor cells [36] and that the combination of nutlin3a with imatinib enhanced CML apoptosis [37]. In addition, p53 stabilization with the MDM2 inhibitor MI-219 was shown to induce apoptosis in BC CML cells [38]. These studies suggest the potential for p53 activation by inhibition of MDM2 as a novel CML therapy, and a potential therapeutic benefit of p53 activation alone or as a sensitizer to other therapeutic agents.

In this study, we examined the expression of p53 and MDM2 in BC CML cells, including proliferating and quiescent CD34^+^ CML progenitor cells, and assessed the effects of nutlin3a and its combination with the Bcl-2 inhibitor ABT-737 and the TKI nilotinib on the viability of these cells. Given that mesenchymal stromal cells (MSCs) in the bone marrow (BM) microenvironment are known to protect leukemia progenitor cells from chemotherapeutic agents [39], we also treated the BC CML cells that were co-cultured with MSCs. We demonstrate that activation of p53 via nutlin3a-induced MDM2 blockade triggers apoptosis in BC CML, including in CD34^+^38^−^ cells and in TKI-insensitive, quiescent CD34^+^ CML progenitor cells. Our findings suggest that MDM2 inhibition acts synergistically with ABT-737 and nilotinib, even in the presence of MSCs, at least in part by regulating the expression of Bcl-2 family proteins.

## RESULTS

### p53 and MDM2 are variably expressed in samples from patients with BC CML

**Figure 1:**
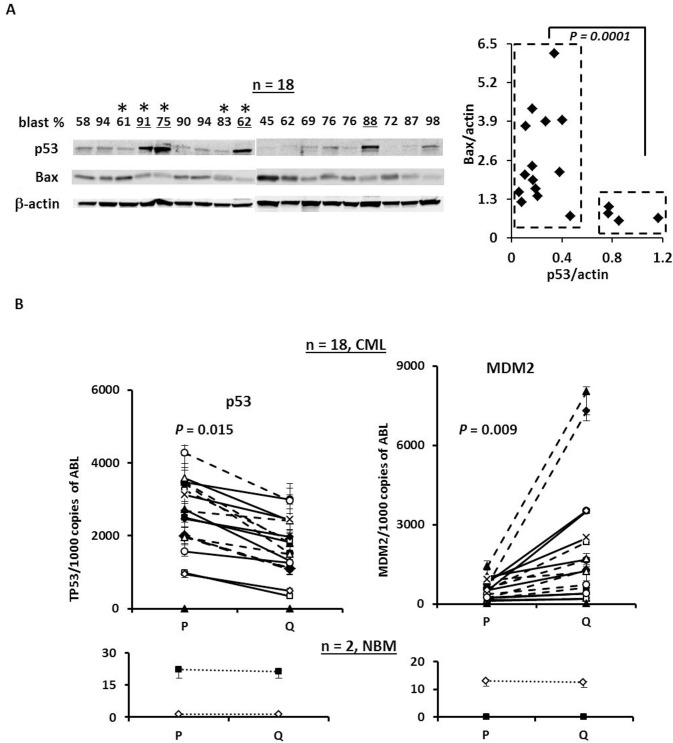
The expression of p53 and MDM2 in samples from BC CML patients **A.** Expression of p53 and Bax in blast cells obtained from BC CML patients by western blot. *, TP53 mutation status was determined by cDNA sequencing. **B.** Expression of p53 and MDM2 in CD34^+^ proliferating and quiescent cells in samples obtained from patients with BC CML and normal controls by real-time RT-PCR. P, proliferating and Q, quiescent.

To test the therapeutic potential of p53 activation by nutlin3a in BC CML, we first examined the expression of p53 using previously stored mononuclear cell lysates isolated from samples obtained from patients with BC CML by western blot. We found that the majority of the samples expressed detectable basal levels of p53 protein (Figure 1A). Four out of eighteen samples (underlined) expressed high basal levels of p53 but significantly lower levels of Bax (Figure 1A) that may indicate *TP53* mutations. To test this, we sequenced *TP53* in the above-referenced samples that had available cDNA (e.g., marked with * in Figure 1A). To our surprise, no *TP53* hot-spot mutations were detected in these samples. We next determined the RNA levels of p53 and MDM2 in proliferating and quiescent CD34^+^ CML progenitor cells by RT-PCR. Of 18 samples, quiescent CD34^+^ cells expressed significantly lower p53 RNA (*P* = 0.015) and higher MDM2 RNA (*P* = 0.009) than the proliferating CD34^+^ cells. This pattern was not observed in RNA derived from normal BM samples (Figure 1B).

### MDM2 inhibition induces apoptosis in bulk and CD34^+^CD38^−^ cells, and sensitizes them to ABT-737 and nilotinib

**Figure 2:**
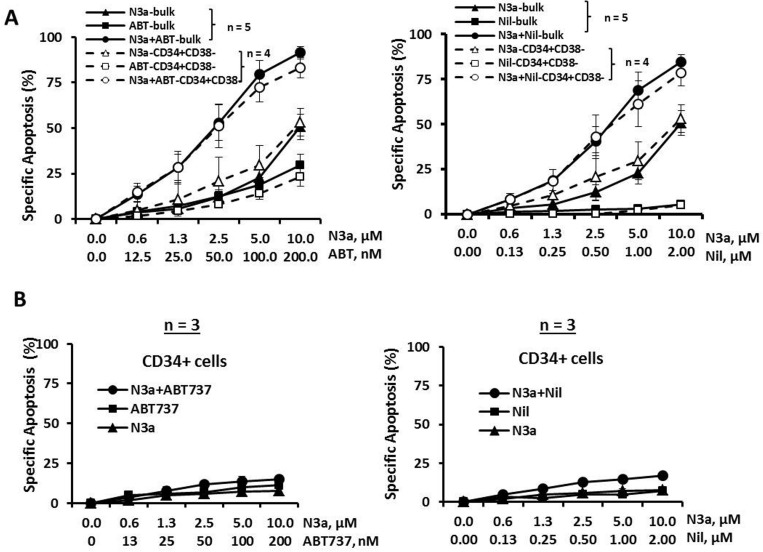
Activation of p53 by nutlin3a induces apoptosis in bulk and CD34^+^CD38^−^ CML cells, synergizes with ABT-737 and nilotinib, and has minimal toxicity to normal CD34^+^ cells Blast cells from BC CML patients or cells from normal BM controls were treated with nutlin3a, ABT-737, nilotinib, nutlin3a plus ABT-737, or nutlin3a plus nilotinib for 48 h. Apoptosis induction was determined in bulk and CD34^+^CD38^−^ CML cells **A.** or CD34^+^ cells from normal BM controls **B.** by flow cytometry after cells were stained with annexin V. N3a, nutlin3a; ABT, ABT-737; and Nil, nilotinib.

Mononuclear cells from BC CML patients who were resistant to multiple TKIs (samples 4, 5, 7, 8b, and 9, Table 1) were treated with nutlin3a and cell death was determined at 48 h. Nutlin3a was able to induce cell death in bulk and CD34^+^CD38^−^ cells (i.e., phenotypically similar as hematopoietic stem/progenitor cells) in all samples analyzed (Figure 2A, triangle lines, *n* = 5 for bulk and *n* = 4 for CD34^+^CD38^−^ cells). When nutlin3a was combined with ABT-737 or nilotinib, apoptosis was greatly enhanced even though the cells were resistant to nilotinib alone (Figure 2A and Table 1). The CI values for the nutlin3a + ABT-737 combination were 0.19 ± 0.06 and 0.62 ± 0.11 for bulk and CD34^+^CD38^−^ cells, respectively and for the nutlin3a + nilotinib combination 0.22 ± 0.05 and 0.36 ± 0.06 for bulk and CD34^+^CD38^−^ cells, respectively. Importantly, nutlin3a alone or in combination with ABT-737 or nilotinib had a minimal activity against CD34^+^ cells from normal BM controls (Figure 2B, *n* = 3).

**Table 1:** Patient characteristics and *in vitro* treatment

Patient #	Source	Blast %	Bcr-Abl Status	Treatment/ Response	*In Vitro* Treatment	*In Vitro C*ell Death/Protein/RNA Analysis	% CD34^+^ Q Cells
# 1	PB	73	T315I	Failed imatinib, dasatinib, and nilotinib; progressed on PHA-739358.	N3a, ABT	P/Q	8.7
# 2	PB	75	T315I, E255K	Failed imatinib and nilotinib.	N3a, ABT	P/Q	1.7
# 3	PB	25	No Mutation	Failed imatinib, treated with nilotinib.	N3a, ABT, Nilotinib	P/Q WB, RT-PCR	4.4
# 4	PB	91	T315I, E255K	Resistance to imatinib; treated with fludarabine, cytarabine, idarubicin, and cytarabine plus dasatinib.	N3a, ABT, Nilotinib	P/Q, Bulk,CD34^+^CD38^−^ WB, PR-PCR,	2.9
# 5	PB	89	T315I, E255V	Failed imatinib, dasatinib, nilotinib, and ponatinib.	N3a, ABT, Nilotinib	P/Q, Bulk,CD34^+^CD38^−^ WB, RT-PCR,	3
# 6	PB	11	No Mutation	Failed imatinib, dasatinib, nilotinib, and ponatinib; treat with decitabine and dasatinib and bosutinib.	N3a, ABT, Nilotinib	P/Q	14.1
# 7	PB	83	H396R	Failed imatinib, dasatinib, nilotinib; treated with DCC-2036 and hydroxyurea.	N3a, ABT, Nilotinib	P/Q, Bulk,CD34^+^CD38^−^	24
# 8a	PB	62	T315I, E255V	Failed imatinib, dasatinib, ponatinib; treated with nilotinib; treat with decitabine and dasatinib.	N3a, ABT, Nilotinib	P/Q, WB, RT-PCR	8
#8b	BM	86	T315I	Failed imatinib, dasatinib, nilotinib and ponatinib; treated with chemotherapy and dual TKI, treated with fludarabine, cytarabine with ponatinib and dasatinib.	N3a, ABT, Nilotinib	Bulk, CD34^+^CD38	
#9	PB	80	No Mutation	Failed imatinib, dasatinib,	N3a, ABT, Nilotinib	Bulk	

**N3a, nutlin3a; ABT, ABT-737; WB, west blot; P, proliferating; Q, quiescent.**

### MDM2 inhibition induces apoptosis in primitive CD34^+^ proliferating and quiescent CML cells from BC CML patients and sensitizes to ABT-737 and nilotinib

**Figure 3:**
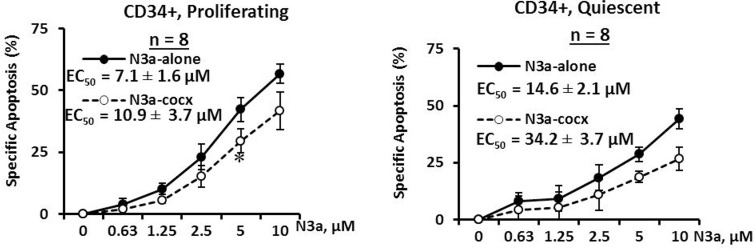
Activation of p53 by nutlin3a induces cell death in both proliferating and quiescent CD34^+^ progenitor cells from patients with BC CML cultured alone or co-cultured with MSCs CSFE stained CML cells were treated with nutlin3a for 48 h with or without MSC co-culture. Apoptotic cell death in CD34^+^ primitive CML cells was determined in proliferating (CSFE^dim^) and quiescent (CSFE^bright^) cells after cells were stained with CD34 antibody and annexin V in 7AAD negative cells. N3a, nutlin3a and cocx, co-culture. *, *P* < 0.05.

To assess the effect of activation of p53 and its ability to sensitize other agents in eliminating quiescent CD34^+^ CML cells, mononuclear cells from BC CML patient samples (#1-8a, Table 1) were stained with the cell division tracking dye CFSE and then co-cultured with human BM derived MSCs. Once proliferating and quiescent cells were distinguishable by flow cytometry, cells were treated with nutlin3a for 48 h with or without MSC co-culture. We found 1.7 to 24.0% CD34^+^ cells were quiescent (Table 1). Nutlin3a was able to induce apoptosis and decrease viable cell counts not only in proliferating (EC_50_ = 7.1 ± 1.6 μM) but also in quiescent (EC_50_ = 14.6 ± 2.1 μM) CD34^+^ CML cells obtained from BC CML patients (Figure 3, *n* = 8, Table 1). Although co-culture with MSCs partially protected leukemic cells from nutlin3a-induced cell death, nutlin3a was still able to induce apoptosis in both proliferating (EC_50_ = 10.9 ± 3.7 μM) and quiescent (EC_50_ = 34.2 ± 3.7 μM) CD34^+^ CML cells (Figure 3).

**Figure 4:**
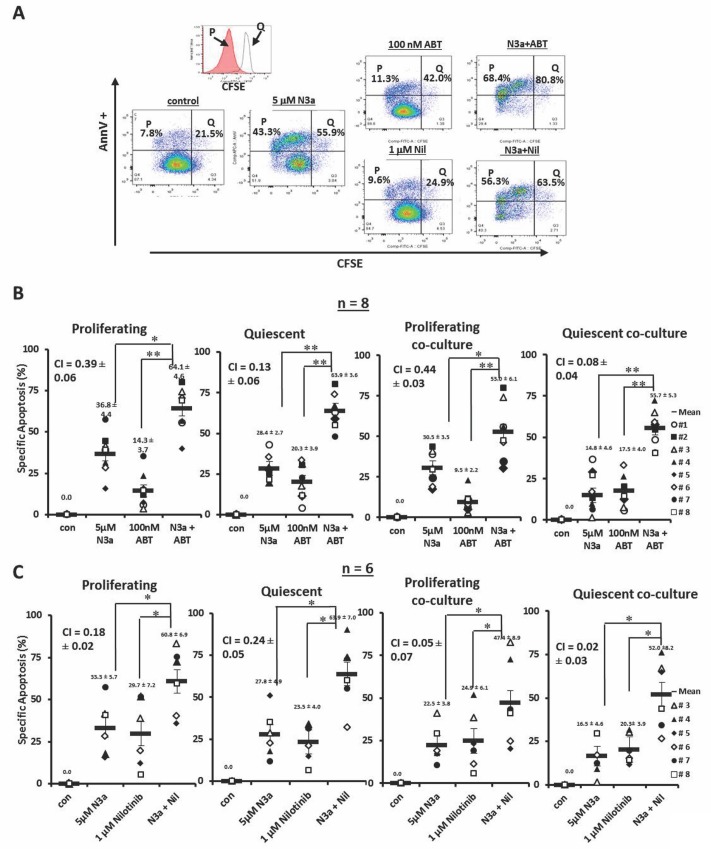
Activation of p53 sensitizes with ABT-737 and nilotinib in both proliferating and quiescent CD34^+^ progenitor cells from patients with BC CML cultured alone or co-cultured with MSCs **A.** CFSE stained cells from a CML patient were treated with nutlin3a, ABT-737, nilotinib, nultin3a plus ABT-737, or nutlin3a plus nilotinib. **B.** CFSE stained cells were treated with nutlin3a, ABT-737, and nutlin3a plus ABT-737 (*n* = 8) or **C.** with nutlin3a, nilotinib, and nutlin3a plus nilotinib (*n* = 6) with or without MSC co-culture. Cell death was determined at 48 h in CD34^+^ proliferating and quiescent cells after cells were stained with annexin V. N3a, nutlin3a; ABT, ABT-737; Nil, nilotinib; cocx, co-culture; *P*, proliferating; and Q, quiescent. *, *P* < 0.05 and **, *P* < 0.001.

CFSE stained cells were treated with nutlin3a, ABT-737, nilotinib, nutlin3a plus ABT-737, or nutlin3a plus nilotinib. A representative result (patient 3) is shown in Figure 4A. Although ABT-737 at low nM was not very active by itself, when combined with nutlin3a, synergistically increased apoptosis in proliferating (CI = 0.39 ± 0.06) and even more so in quiescent (CI = 0.13 ± 0.06) CD34^+^ CML BC cells. This held up even when cells were co-cultured with MSCs (CI = 0.44 ± 0.03 for proliferating and CI = 0.08 ± 0.04 for quiescent CD34^+^ cells) (Figure 4B, *n* = 8, Table 1). Similar results were obtained when nutlin3a was combined with nilotinib in proliferating (CI = 0.18 ± 0.02 without and CI = 0.05 ± 0.07 with co-culture) and in quiescent CD34^+^ cells (CI = 0.24 ± 0.05 without and CI = 0.02 ± 0.03 with co-culture) (Figure 4C, *n* = 6, Table 1).

### Modulation of pro-apoptotic and anti-apoptotic Bcl-2 proteins as a mechanism of the synergistic action of MDM2-inhibitor combinations

**Figure 5:**
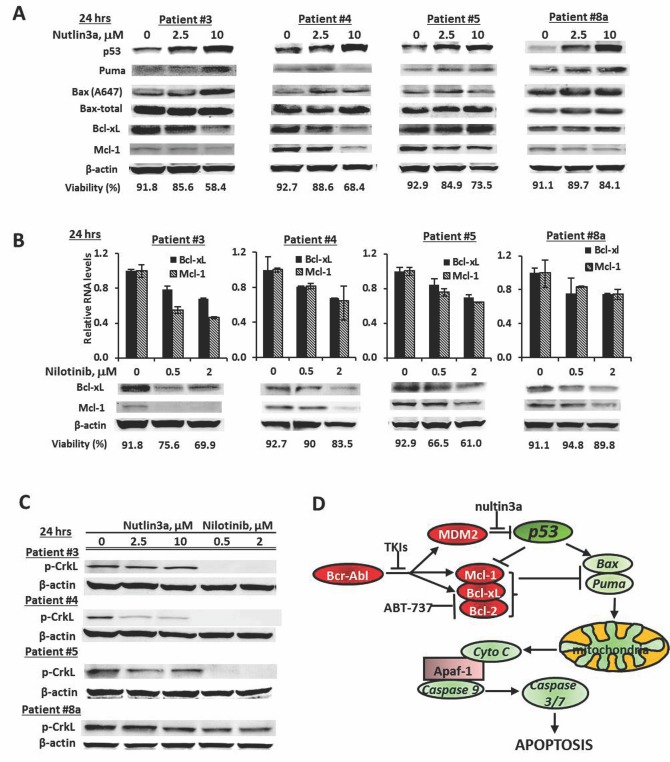
Induction of pro-apoptotic and suppression of anti-apoptotic Bcl-2 family proteins as mechanisms of synergy **A.** Cells from BC CML samples were treated with nutlin3a for 24 h and protein levels were determined by western blot. **B.** Cells from BC CML samples were treated with nilotinib for 24 h. RNA levels were determined by real time RT-PCR and protein levels by western blot. Cell viability was shown at the bottom of each graph. **C.** Cells from BC CML samples were treated with nutlin3a or nilotinib for 24 h. p-CrkL levels were determined by western blot. **D.** A schema representing the mechanisms of synergistic cell killing by nutlin, ABT-737, and nilotinib.

To better understand the mechanism(s) underlying the observed synergistic effect, we treated mononuclear cells from CML patients (Table 1, sample 3, 4, 5, and 8a) with nutlin3a and nilotinib. Nutlin3a induced p53 in all samples tested and increased expression of the pro-apoptotic Bcl-2 protein Puma, and/or of the active form of Bax (A647) indicating activation of p53 transcriptional activity. No consistent increase of Noxa or Bim was observed (not shown), although all the samples showed synergistic responses to the nutlin3a and ABT-737 combination (Figure 4B) suggesting that Noxa and Bim induction was not a determinant factor for the synergy. In addition, nutlin3a decreased levels of the anti-apoptotic proteins Bcl-xL, Mcl-1 or both (Figure 5A). Nilotinib also decreased the expression of Mcl-1 and Bcl-xL, both at the RNA and protein levels (Figure 5B), even in patients who had not responded to nilotinib treatment clinically (Table 1). To demonstrate that decreased Mcl-1 and Bcl-xL expression is at least in part mediated through nilotinib-induced Bcr-Abl inhibition, we determined p-CrkL levels as indicators of Bcr-Abl signaling and found that p-CrkL was greatly decreased in samples 3, 4, and 5 and partially decreased in sample 8a, while nutlin3a in general did not affect Bcr-Abl signaling (Figure 5C). We also observed decreased MDM2 levels in nilotinib treated cells (not shown). ABT-737 is a known inhibitor for Bcl-2 and Bcl-xL. Therefore, nutlin3a and ABT-737 or nutlin3a and nilotinib combinations both cooperatively modulate Bcl-2 family proteins by inducing pro- and inhibiting anti-apoptotic Bcl-2 family members (Figure 5D).

## DISCUSSION

We report in this study that p53 is variably expressed in cells from BC CML patient samples, and that MDM2 expression is significantly higher, and p53 expression is lower, in quiescent compared to proliferating CD34^+^ progenitor cells. Activation of p53 was shown to induce apoptosis in bulk and CD34^+^CD38^−^ BC CML cells, as well as in proliferating and, most importantly, in TKI-insensitive, CD34^+^ quiescent BC CML progenitor cells. In addition, p53 activation in combination with inhibition of Bcl-2 or Bcr-Abl synergistically induced cell death in these cells. This effect was achieved in part by concomitantly inducing pro-apoptotic and suppressing anti-apoptotic Bcl-2 proteins. In agreement with this notion, our group has reported that nutlin3a and ABT-737 combination synergistically induces apoptosis in AML cells in part by inducing the active form of Bax [40]. Nutlin3a alone or in combinations with ABT-737 or nilotinib was shown to have limited toxicity in normal CD34^+^ BM cells.

Low basal levels of p53 were observed in the majority (14/18) of PB or BM samples from BC CML patients. Sequence analysis did not find hot-spot mutations in *TP53* even in samples with high p53 expression and low Bax expression. Interestingly, we found the P72R mutation in most samples analyzed. This mutation was reported to maintain p53 activity but its functional significance is unclear [41]. These data suggest that most of the patients carry wild-type p53; thus, pharmacological activation of p53 should be a feasible therapeutic strategy for this disease. This concept is further supported by our results showing that treating samples obtained from BC CML patients with nutlin3a activates p53 and induces cell death. The mechanisms of lower p53 and higher MDM2 expression levels in quiescent as compared to proliferating CD34^+^ CML cells are unknown at the present time. However, Bcl-6-mediated p53 repression in CML stem cells has been reported [42]. We also observed higher Bcl-6 levels in quiescent compared to proliferating CML progenitor cells (result not shown), but defining and validating this mechanism requires further study.

We and others have reported that overexpression of MDM2 correlated with nutlin3a sensitivity in both AML and ALL [28, 32]. Although we observed significantly higher MDM2 levels in quiescent compared to proliferating CD34^+^ cells, we found lower, rather than higher, sensitivity of quiescent CD34^+^ CML cells to nutlin3a, compared to proliferating cells. Many factors can explain this finding such as lower expression levels of p53 in quiescent CD34^+^ CML cell populations, or a higher Bcr-Abl kinase activity [14, 43] that may activate alternative survival pathways.

Although MSCs are known to protect leukemia cells from various chemotherapeutic agent-induced apoptosis, these cells only partially abrogated the cytotoxic effects of nutlin3a and nultin3a in combinations with either ABT-737 or nilotinib on the CML cells. We previously reported that activation of p53 by nutlin3a suppressed CXCL-12 expression and CXCL12 secretion by 70% [44]. CXCR4/CXCL12-mediated leukemia/stromal interactions are known to contribute to chemoresistance in CML [45–47] and are greatly diminished if CXCL12 concentration or activity is reduced.

Nutlin3a and its combination with ABT-737 and nilotinib both induced apoptosis in bulk and in CD34^+^CD38^−^ BC cells with similar efficacy. A sub-population of CD34^+^ cells is quiescent. This population has been shown to be able to engraft NOD/SCID mice and initiate CML, is insensitive to various therapeutic agents including TKIs, and is responsible for disease recurrence after ceasing TKI therapy even in patients who had achieved molecular responses [7, 8]. We found that 1.7 to 24% of CD34^+^ cells from BC CML patient samples analyzed were quiescent. Most importantly, nutlin3a induced apoptosis in both, proliferating and quiescent CD34^+^ BC CML cells. This apoptosis induction was further enhanced by combining nutlin3a with ABT-737 and nilotinib even in cells from TKI-resistance/relapsed patients under physiologic MSC co-culture conditions. We observed that more cell death was induced in bulk and CD34^+^CD38^−^ (Figure 2) than CD34^+^ proliferating and quiescent CML cells (Figure 4) under the same conditions, probably because the latter had been cultured with MSCs for 5-12 d before therapy to distinguish proliferating and quiescent cells.

We also observed that nutlin3a decreased Bcl-xL and Mcl-1 protein levels. This effect is likely related to cleavage of these proteins by activated caspases as the decreases are more obvious in patients 3 and 4 which showed lower cell viability. CML cells develop resistance to TKIs through multiple mechanisms that are both Bcl-Abl dependent and independent. Although clinically not responsive to TKIs including nilotinib, all samples treated with nilotinib showed inhibition of Bcr-Abl signaling as measured by reduction of p-CrkL and decrease of the Bcr-Abl downstream target proteins Mcl-1 and Bcl-xL. We speculate that partial suppression of Mcl-1 and Bcl-xL by nilotinib was likely sufficient to lower the apoptotic threshold and sensitize CML cells to nutlin3a-induced cell death, although the mechanism associated with nilotinib's activity in the Bcr-Abl-mutant samples remains to be fully elucidated. ABT-737 is a known Bcl-2/Bcl-xL inhibitor [48, 49]. By simultaneously targeting various arms of Bcl-2 regulation (as shown in Figure 5D), we were able to synergistically induce apoptosis in bulk, CD34^+^CD38^−^, and CD34^+^ proliferating and quiescent progenitor cells from samples obtained from patients with BC CML even when cells were not sensitive to a single agent or co-cultured with MSCs. Interestingly, the degree of drug sensitivity in each patient sample was not consistent to each treatment (each of single drug or combinations), under different culture conditions (with or without co-culture), or in different cell compartments (proliferating or quiescent cells) suggesting that each sample responded differently to each drug and their combinations, was affected differently by the stromal cells, or had different molecular signatures in proliferating and quiescent cell populations. Nevertheless, the combinations were more effective in apoptosis induction in all patient samples, without or with co-cultures, and in both proliferating and quiescent CD34^+^ progenitor cell populations.

In conclusion, combinations of nutlin3a with ABT-737 or with nilotinib cooperatively targeted various determinants of Bcl-2-mediated apoptosis. Activation of p53 by blockade of MDM2 sensitized BC CML cells, including CD34^+^CD38^−^ and quiescent CD34^+^ progenitor cells, to TKI- and Bcl-2 inhibitor-induced apoptosis but had very limited toxicity in CD34^+^ cells from normal BM controls, suggesting a potential for utilizing this strategy in the treatment of BC CML and elimination of quiescent CML stem/progenitor cells. TKIs are front-line therapies for CML. MDM2 inhibitors and Bcl-2 inhibitors are in clinical trials; hence, their combinations are feasible strategies and could quickly move into clinical trials.

## MATERIALS AND METHODS

### Cells and cell treatment

Samples from BC CML patients or normal controls were acquired after informed consent following IRB approved study protocols and in concordance with the declaration of Helsinki. Patient characteristics are shown in Table 1. Mononuclear cells, isolated by Ficoll-Hypaque density-gradient centrifugation from patients and normal controls were treated with various concentrations of nutlin3a, ABT-737, nilotinib, nutlin3a plus ABT-737, or nutlin3a plus nilotinib (Table 1). MSCs were isolated from BM of healthy subjects as previously described [30, 50].

### Identification of proliferating and quiescent CML cells

Mononuclear cells from CML patients (Table 1) were stained with 5-(and 6-) carboxy-fluorescein diacetate succinimidyl ester (CFSE, Life Technologies, Carlsbad, CA) as described [51]. They were then co-cultured with MSCs in αMEM /10%FCS medium [13]. After proliferating (CFSE^dim^) and quiescent (CFSE^bright^) cells became distinguishable, cells were either stained with CD34 antibody (BD Biosciences, San Jose, CA) and sorted (BD FACSAria^TM^ II sorter, BD Biosciences) into proliferating or quiescent fractions [14] or treated with nutlin3a, ABT-737, nilotinib, nutlin3a plus ABT-737, or nutlin3a plus nilotinib (Table 1) with or without MSCs.

### Apoptosis assay

Apoptosis in bulk and CD34^+^CD38^−^ cells was estimated by flow cytometric measurements of annexin V stained cells using a BD FACSArray Bioanalyzer. CFSE stained cells were incubated with CD34 antibody and 7-amino-actinomycin D (7AAD). Apoptosis was estimated in 7AAD^−^ CML progenitor cells using a BD LSR-II flow cytometer. Apoptotic quiescent or proliferating CML progenitor cells were defined as annexin V positivity in the CD34^+^CFSE^bright^ or CD34^+^CFSE^dim^ population. Apoptotic leukemia cells co-cultured with MSCs are defined as annexin V+/CD45^+^ cells. Apoptosis was expressed as specific apoptosis [14]:

$$\begin{array}{ccc} \frac{\%\text{ of apoptosis in treated cells}-\;\%\text{ of apoptosis in untreated cells}}{\%\text{ of visible untreated cells}} & \times & 100\% \end{array}$$

### Western blot

Protein levels were determined by western blot as described previously [14, 43]. Antibodies against p53 (#SC-126), MDM2 (#SC-5304), and Mcl-1 (#SC-819) were purchased from Santa Cruz Biotechnology Inc. (Dallas, TX); Bax (#554104) from BD Pharmingen (San Jose, CA); Bax (A647) (#B-8429) from Sigma (St. Louis, MO); Puma (ab9643) from Abcam (Cambridge, MS); Bcl-2 (#M0887) from Dako (Carpentaria, CA); and Bcl-xL (#2762s) and p-CrkL (#3181s) from Cell Signaling Technology (Danvers, MA). β-Actin (#A5316) from Sigma was used as a loading control.

### Real-time RT-PCR

RT-PCR was carried out as previously described [14, 43]. TaqMan primer sets for Bcl-xL (Hs00236329_m1), Mcl-1 (Hs03043899_m1), MDM2 (Hs00242813_m1), p53 (Hs99999147_m1), and ABL1 (Hs01104728_m1) were obtained from Applied Biosystems (Foster City, CA). The abundance of each transcript relative to that of ABL1 was calculated using the 2^−ΔCt^ method, where ΔCt is the mean Ct of the transcript of interest minus the mean Ct of the transcript for ABL1.

### TP53 mutational analysis

*TP53* was sequenced using the primers and the method described previously with modifications [52]. Briefly, the full length open reading frame cDNA was amplified using Q5 Hot Start High Fidelity DNA polymerase (New England Biolabs, Beverly, MA) as recommended by the manufacturer. Direct sequencing on both strands was performed using the dideoxy method with BigDye terminators. Primers were obtained from Integrated DNA Technologies (Coralville, IA), and sequencing analysis was performed by Lone Star Labs (Houston, TX).

### Statistical analyses

Multiple samples were treated and the results were expressed as the mean ± standard error of the mean (SEM). Statistical significance was set at *P* < 0.05, using two-sided Student's *t*-test. EC_50_ was calculated by Calcusyn software. The combination index (CI), determined using the Chou-Talalay method [53] and Calcusyn software, was expressed as the mean of CI values obtained at ED_50_, ED_75_, and ED_90_. CI < 1 was considered synergistic. PCR was done in duplicates and results expressed as mean ± standard derivation.

## References

[1] Sawyers CL, Hochhaus A, Feldman E, Goldman JM, Miller CB, Ottmann OG, Schiffer CA, Talpaz M, Guilhot F, Deininger MW, Fischer T, O'Brien SG, Stone RM (2002). Imatinib induces hematologic and cytogenetic responses in patients with chronic myelogenous leukemia in myeloid blast crisis: results of a phase II study. Blood.

[2] Copland M, Hamilton A, Elrick LJ, Baird JW, Allan EK, Jordanides N, Barow M, Mountford JC, Holyoake TL (2006). Dasatinib (BMS-354825) targets an earlier progenitor population than imatinib in primary CML, but does not eliminate the quiescent fraction. Blood.

[3] Corbin AS, Agarwal A, Loriaux M, Cortes J, Deininger MW, Druker BJ (2011). Human chronic myeloid leukemia stem cells are insensitive to imatinib despite inhibition of BCR-ABL activity. J Clin Invest.

[4] Graham SM, Jorgensen HG, Allan E, Pearson C, Alcorn MJ, Richmond L, Holyoake TL (2002). Primitive, quiescent, Philadelphia-positive stem cells from patients with chronic myeloid leukemia are insensitive to STI571 in vitro. Blood.

[5] Heaney NB, Holyoake TL (2007). Therapeutic targets in chronic myeloid leukaemia. Hematol Oncol.

[6] Holtz MS, Forman SJ, Bhatia R (2005). Nonproliferating CML CD34+ progenitors are resistant to apoptosis induced by a wide range of proapoptotic stimuli. Leukemia.

[7] Elrick LJ, Jorgensen HG, Mountford JC, Holyoake TL (2005). Punish the parent not the progeny. Blood.

[8] Holyoake T, Jiang X, Eaves C, Eaves A (1999). Isolation of a highly quiescent subpopulation of primitive leukemic cells in chronic myeloid leukemia. Blood.

[9] Ross DM, Branford S, Seymour JF, Schwarer AP, Arthur C, Yeung DT, Dang P, Goyne JM, Slader C, Filshie RJ, Mills AK, Melo JV, White DL (2013). Safety and efficacy of imatinib cessation for CML patients with stable undetectable minimal residual disease: results from the TWISTER study. Blood.

[10] Jamieson CH, Ailles LE, Dylla SJ, Muijtjens M, Jones C, Zehnder JL, Gotlib J, Li K, Manz MG, Keating A, Sawyers CL, Weissman IL (2004). Granulocyte-macrophage progenitors as candidate leukemic stem cells in blast-crisis CML. N Engl J Med.

[11] Savona M, Talpaz M (2008). Getting to the stem of chronic myeloid leukaemia. Nat Rev Cancer.

[12] Goff DJ, Court Recart A, Sadarangani A, Chun HJ, Barrett CL, Krajewska M, Leu H, Low-Marchelli J, Ma W, Shih AY, Wei J, Zhai D, Geron I (2013). A Pan-BCL2 inhibitor renders bone-marrow-resident human leukemia stem cells sensitive to tyrosine kinase inhibition. Cell Stem Cell.

[13] Mak DH, Schober WD, Chen W, Konopleva M, Cortes J, Kantarjian HM, Andreeff M, Carter BZ (2009). Triptolide induces cell death independent of cellular responses to imatinib in blast crisis chronic myelogenous leukemia cells including quiescent CD34+ primitive progenitor cells. Mol Cancer Ther.

[14] Mak DH, Wang RY, Schober WD, Konopleva M, Cortes J, Kantarjian H, Andreeff M, Carter BZ (2012). Activation of apoptosis signaling eliminates CD34+ progenitor cells in blast crisis CML independent of response to tyrosine kinase inhibitors. Leukemia.

[15] Reddiconto G, Toto C, Palama I, De Leo S, de Luca E, De Matteis S, Dini L, Passerini CG, Di Renzo N, Maffia M, Coluccia AM (2012). Targeting of GSK3beta promotes imatinib-mediated apoptosis in quiescent CD34+ chronic myeloid leukemia progenitors, preserving normal stem cells. Blood.

[16] Zhang B, Li M, McDonald T, Holyoake TL, Moon RT, Campana D, Shultz L, Bhatia R (2013). Microenvironmental protection of CML stem and progenitor cells from tyrosine kinase inhibitors through N-cadherin and Wnt-beta-catenin signaling. Blood.

[17] Zhang B, Strauss AC, Chu S, Li M, Ho Y, Shiang KD, Snyder DS, Huettner CS, Shultz L, Holyoake T, Bhatia R (2010). Effective targeting of quiescent chronic myelogenous leukemia stem cells by histone deacetylase inhibitors in combination with imatinib mesylate. Cancer Cell.

[18] Aichberger KJ, Mayerhofer M, Krauth MT, Skvara H, Florian S, Sonneck K, Akgul C, Derdak S, Pickl WF, Wacheck V, Selzer E, Monia BP, Moriggl R (2005). Identification of mcl-1 as a BCR/ABL-dependent target in chronic myeloid leukemia (CML): evidence for cooperative antileukemic effects of imatinib and mcl-1 antisense oligonucleotides. Blood.

[19] Horita M, Andreu EJ, Benito A, Arbona C, Sanz C, Benet I, Prosper F, Fernandez-Luna JL (2000). Blockade of the Bcr-Abl kinase activity induces apoptosis of chronic myelogenous leukemia cells by suppressing signal transducer activator of transcription 5-dependent expression of Bcl-xL. J Exp Med.

[20] Kuroda J, Kimura S, Andreeff M, Ashihara E, Kamitsuji Y, Yokota A, Kawata E, Takeuchi M, Tanaka R, Murotani Y, Matsumoto Y, Tanaka H, Strasser A (2008). ABT-737 is a useful component of combinatory chemotherapies for chronic myeloid leukaemias with diverse drug-resistance mechanisms. Br J Haematol.

[21] Kuroda J, Kimura S, Strasser A, Andreeff M, O'Reilly LA, Ashihara E, Kamitsuji Y, Yokota A, Kawata E, Takeuchi M, Tanaka R, Tabe Y, Taniwaki M (2007). Apoptosis-based dual molecular targeting by INNO-406, a second-generation Bcr-Abl inhibitor, and ABT-737, an inhibitor of antiapoptotic Bcl-2 proteins, against Bcr-Abl-positive leukemia. Cell Death Differ.

[22] Miyashita T, Reed JC (1995). Tumor suppressor p53 is a direct transcriptional activator of the human bax gene. Cell.

[23] Vogelstein B, Lane D, Levine AJ (2000). Surfing the p53 network. Nature.

[24] Yu J, Zhang L (2003). No PUMA, no death: implications for p53-dependent apoptosis. Cancer Cell.

[25] Vaseva AV, Moll UM (2009). The mitochondrial p53 pathway. Biochim Biophys Acta.

[26] Green DR, Kroemer G (2009). Cytoplasmic functions of the tumour suppressor p53. Nature.

[27] Moll UM, Petrenkol O (2003). The MDM2-p53 interaction. Mol Cancer Res.

[28] Kojima K, Konopleva M, Samudio IJ, Shikami M, Cabreira-Hansen M, McQueen T, Ruvolo V, Tsao T, Zeng Z, Vassilev LT, Andreeff M (2005). MDM2 antagonists induce p53-dependent apoptosis in AML: implications for leukemia therapy. Blood.

[29] Kojima K, Konopleva M, Tsao T, Nakakuma H, Andreeff M (2008). Concomitant inhibition of Mdm2-p53 interaction and Aurora kinases activates the p53-dependent postmitotic checkpoints and synergistically induces p53-mediated mitochondrial apoptosis along with reduced endoreduplication in acute myelogenous leukemia. Blood.

[30] Kojima K, Konopleva M, McQueen T, O'Brien S, Plunkett W, Andreeff M (2006). Mdm2 inhibitor Nutlin-3a induces p53-mediated apoptosis by transcription-dependent and transcription-independent mechanisms and may overcome Atm-mediated resistance to fludarabine in chronic lymphocytic leukemia. Blood.

[31] Coll-Mulet L, Iglesias-Serret D, Santidrian AF, Cosialls AM, de Frias M, Castano E, Campas C, Barragan M, de Sevilla AF, Domingo A, Vassilev LT, Pons G, Gil J (2006). MDM2 antagonists activate p53 and synergize with genotoxic drugs in B-cell chronic lymphocytic leukemia cells. Blood.

[32] Gu L, Zhu N, Findley HW, Zhou M (2008). MDM2 antagonist nutlin-3 is a potent inducer of apoptosis in pediatric acute lymphoblastic leukemia cells with wild-type p53 and overexpression of MDM2. Leukemia.

[33] Beck Z, Kiss A, Toth FD, Szabo J, Bacsi A, Balogh E, Borbely A, Telek B, Kovacs E, Olah E, Rak K (2000). Alterations of P53 and RB genes and the evolution of the accelerated phase of chronic myeloid leukemia. Leuk Lymphoma.

[34] Jiang Q, Crews LA, Barrett CL, Chun HJ, Court AC, Isquith JM, Zipeto MA, Goff DJ, Minden M, Sadarangani A, Rusert JM, Dao KH, Morris SR (2013). ADAR1 promotes malignant progenitor reprogramming in chronic myeloid leukemia. Proc Natl Acad Sci U S A.

[35] Goetz AW, van der Kuip H, Maya R, Oren M, Aulitzky WE (2001). Requirement for Mdm2 in the survival effects of Bcr-Abl and interleukin 3 in hematopoietic cells. Cancer Res.

[36] Li L, Wang L, Wang Z, Ho Y, McDonald T, Holyoake TL, Chen W, Bhatia R (2012). Activation of p53 by SIRT1 inhibition enhances elimination of CML leukemia stem cells in combination with imatinib. Cancer Cell.

[37] Kurosu T, Wu N, Oshikawa G, Kagechika H, Miura O (2010). Enhancement of imatinib-induced apoptosis of BCR/ABL-expressing cells by nutlin-3 through synergistic activation of the mitochondrial apoptotic pathway. Apoptosis.

[38] Peterson LF, Mitrikeska E, Giannola D, Lui Y, Sun H, Bixby D, Malek SN, Donato NJ, Wang S, Talpaz M (2011). p53 stabilization induces apoptosis in chronic myeloid leukemia blast crisis cells. Leukemia.

[39] Konopleva M, Tabe Y, Zeng Z, Andreeff M (2009). Therapeutic targeting of microenvironmental interactions in leukemia: mechanisms and approaches. Drug ResistUpdat.

[40] Kojima K, Konopleva M, Samudio IJ, Schober WD, Bornmann WG, Andreeff M (2006). Concomitant inhibition of MDM2 and Bcl-2 protein function synergistically induce mitochondrial apoptosis in AML. Cell Cycle.

[41] Whibley C, Pharoah PD, Hollstein M (2009). p53 polymorphisms: cancer implications. Nat Rev Cancer.

[42] Hurtz C, Hatzi K, Cerchietti L, Braig M, Park E, Kim YM, Herzog S, Ramezani-Rad P, Jumaa H, Muller MC, Hofmann WK, Hochhaus A, Ye BH (2011). BCL6-mediated repression of p53 is critical for leukemia stem cell survival in chronic myeloid leukemia. J Exp Med.

[43] Carter BZ, Mak DH, Wang Z, Ma W, Mak PY, Andreeff M, Davis RE (2013). XIAP downregulation promotes caspase-dependent inhibition of proteasome activity in AML cells. Leuk Res.

[44] Kojima K, McQueen T, Chen Y, Jacamo R, Konopleva M, Shinojima N, Shpall E, Huang X, Andreeff M (2011). p53 activation of mesenchymal stromal cells partially abrogates microenvironment-mediated resistance to FLT3 inhibition in AML through HIF-1alpha-mediated down-regulation of CXCL12. Blood.

[45] Jin LH, Tabe Y, Konoplev S, Xu YY, Leysath CE, Lu HB, Kimura S, Ohsaka A, Rios MB, Calvert L, Kantarjian H, Andreeff M, Konopleva M (2008). CXCR4 up-regulation by imatinib induces chronic myelogenous leukemia (CML) cell migration to bone marrow stroma and promotes survival of quiescent CML cells. Mol Ca Ther.

[46] Tabe Y, Jin L, Iwabuchi K, Wang RY, Ichikawa N, Miida T, Cortes J, Andreeff M, Konopleva M (2012). Role of stromal microenvironment in nonpharmacological resistance of CML to imatinib through Lyn/CXCR4 interactions in lipid rafts. Leukemia.

[47] Beider K, Darash-Yahana M, Blaier O, Koren-Michowitz M, Abraham M, Wald H, Wald O, Galun E, Eizenberg O, Peled A, Nagler A (2014). Combination of Imatinib with CXCR4 antagonist BKT140 overcomes the protective effect of stroma and targets CML *in vitro* and *in vivo*. Mol Cancer Ther.

[48] Konopleva M, Contractor R, Tsao T, Samudio I, Ruvolo PP, Kitada S, Deng X, Zhai D, Shi YX, Sneed T, Verhaegen M, Soengas M, Ruvolo VR (2006). Mechanisms of apoptosis sensitivity and resistance to the BH3 mimetic ABT-737 in acute myeloid leukemia. Cancer Cell.

[49] Oltersdorf T, Elmore SW, Shoemaker AR, Armstrong RC, Augeri DJ, Belli BA, Bruncko M, Deckwerth TL, Dinges J, Hajduk PJ, Joseph MK, Kitada S, Korsmeyer SJ (2005). An inhibitor of Bcl-2 family proteins induces regression of solid tumours. Nature.

[50] Studeny M, Marini FC, Champlin RE, Zompetta C, Fidler IJ, Andreeff M (2002). Bone marrow-derived mesenchymal stem cells as vehicles for interferon-beta delivery into tumors. Cancer Res.

[51] Holtz MS, Slovak ML, Zhang F, Sawyers CL, Forman SJ, Bhatia R (2002). Imatinib mesylate (STI571) inhibits growth of primitive malignant progenitors in chronic myelogenous leukemia through reversal of abnormally increased proliferation. Blood.

[52] Kato H, Kato S, Kumabe T, Sonoda Y, Yoshimoto T, Kato S, Han SY, Suzuki T, Shibata H, Kanamaru R, Ishioka C (2000). Functional evaluation of p53 and PTEN gene mutations in gliomas. Clin Cancer Res.

[53] Chou TC, Talalay P (1984). Quantitative analysis of dose-effect relationships: the combined effects of multiple drugs or enzyme inhibitors. Adv Enzyme Regul.

